# Abstracts from the 71^st^ Annual Meeting of the British Thyroid Association

**DOI:** 10.1186/s13044-023-00170-8

**Published:** 2023-07-11

## L1 George Murray Lecture: The molecular analysis in thyroid cancer: From a researchers’ curiosity to a clinicians’ need

### Laura Fugazzola^1,2^

#### ^1^Department of Endocrine and Metabolic Diseases, Endocrine Oncology Unit, IRCCS Istituto Auxologico Italiano, Milan, Italy; ^2^Department of Pathophysiology and Transplantation, University of Milan, Milan, Italy

##### **Correspondence:** Laura Fugazzola (laura.fugazzola@unimi.it)

*Thyroid Research 2023*, **16(S1):**L1

Thyroid tumors show a wide range of lesions varying from slowly progressive well-differentiated tumors to anaplastic highly malignant cancers. Therefore, they have always represented an attractive model to investigate the role of oncogene activation in different stages of the neoplastic state. When ret/PTC, the first oncogene unique to malignant forms, was described in 1987 by two Italian groups (Fusco et al., Nature 1987), it was hard to imagine that, 25 years after, that finding would have become crucial in the preoperative diagnosis of thyroid cancer. Moreover, it would have been almost unconceivable to think that, 35 years after, that genetic alteration would have been targeted by specific drugs to be used in patients with advanced and metastatic forms of cancer. Actually, the landscape of genetic alterations has progressively and rapidly increased in the last years and nowadays the majority of thyroid tumors has its own genetic identity. This knowledge put the bases for the creation of panels able to distinguish between benign and malignant cases among indeterminate nodules, allowing to preoperatively identify the best treatment. More recently, again based on the molecular genetic findings, targeted compounds have been tested and approved for the treatment of tumors harboring some of the disease-causing mutations. In the last 3 decades the research in the thyroid cancer field has been full of basic and translational crucial findings, and it has been extremely fascinating to go across this and to participate with some new insights. In this presentation, we will travel through the evolving knowledge in thyroid cancer genetics and its applications in the clinical setting.

## S1 Challenges in Managing Paediatric Thyroid disease - From Infancy to Adolescence

### Renuka Dias

#### Birmingham Women and Children’s Hospital, Birmingham, UK

##### **Correspondence:** Renuka Dias (r.dias1@nhs.net)

*Thyroid Research 2023*, **16(S1):**S1

Management of thyroid disease in children can be challenging and many of the young people we manage will come to adult services. Through a series of case vignettes, I hope to share the range of complexity of thyroid disorders faced by Paediatric Endocrinologists from birth to young adulthood (and everything in between).

## S2 Amiodarone-induced thyroid disorders

### Mark Vanderpump

#### OneWelbeck Endocrinology, London, UK

##### **Correspondence:** Mark Vanderpump (mark.vanderpump@googlemail.com)

*Thyroid Research 2023*, **16(S1):**S2

Amiodarone is a benzofuran effective in the management of supraventricular arrhythmias. Because of its high iodine content (about 37% by weight, with a daily dissociation rate of iodine from the drug of about 10%) and pharmacological properties (inhibition of peripheral mono-deiodination of thyroxine, T4), the drug causes changes in thyroid function tests and may be responsible for thyroid dysfunction. Approximately 15-20% of amiodarone-treated patients develop either thyrotoxicosis (amiodarone-induced thyrotoxicosis, AIT) or hypothyroidism (amiodarone-induced hypothyroidism, AIH). AIH is relatively more frequent in iodine-replete and AIT in iodine-deficient geographical areas. Both AIT and AIH may occur early or late during amiodarone treatment, and develop in an apparently normal thyroid gland or in a gland with pre-existing abnormalities (nodular goitre, latent Graves’ disease, chronic autoimmune thyroiditis). Diagnosis, classification, and management of amiodarone-induced thyroid dysfunction, particularly AIT, are often challenging with limited evidence provided by randomized clinical trials. Both AIH and AIT may develop in apparently normal thyroid glands or in the presence of underlying thyroid abnormalities. Female gender and anti-thyroid peroxidase antibodies seem to predict AIH which does not require amiodarone withdrawal, and is treated with levothyroxine replacement if overt, whereas subclinical forms may be followed without treatment. Two main types of AIT are recognised: type 1 AIT, a form of iodine-induced hyperthyroidism occurring in nodular goitres or latent Graves’ disease, and type 2 AIT, resulting from destructive thyroiditis in a normal thyroid gland. Mixed/indefinite forms exist, due to both pathogenic mechanisms. Type 1 AIT is best treated with thionamides and type 2 AIT is treated with oral glucocorticoids. Once euthyroidism has been restored, type 2 AIT patients are followed without treatment, whereas type 1 AIT patients may require thyroidectomy or radioiodine.

## S3 Subclinical hyperthyroidism

### Bernard Goichot

#### Department of Endocrinology, Hôpital Hautepierre, Strasbourg University Hospital, Strasbourg, France

##### **Correspondence:** Bernard Goichot (bernard.goichot@chru-strasbourg.fr)

*Thyroid Research 2023*, **16(S1):**S3

Subclinical hyperthyroidism (SCH) is a biologically defined condition which can have deleterious effects on health, in particular among the elderly. More than a disease, it may be considered as a potential risk factor for various complications. Numerous publications have reported a possible link between SCH and cardiac endpoints. The most demonstrated association is with atrial fibrillation (AF). However, no randomized clinical trial (RCT) has yet demonstrated any benefits of treating SCH, in particular to prevent the risk of AF.

For the last ten years there have been several guidelines or experts’ opinions published to help patients’ management despite the absence of scientific evidence. Age, degree of TSH suppression (“grade”), co-existing cardiac risk factors and etiology of SCH are generally proposed as key factors that have to be taken into account in the decision to treat or to propose surveillance to the patients. The role of these parameters will be discussed and preliminary results of the French RCT PIRATHES comparing treatment of SCH to surveillance in patients more than 50 years old will be presented.

## S4 Year in basic thyroidology

### Peter Kopp^1,2^

#### ^1^Division of Endocrinology, Diabetology and Metabolism, University of Lausanne, Switzerland; ^2^Division of Endocrinology, Metabolism and Molecular Medicine, Northwestern University, Chicago, USA

##### **Correspondence:** Peter Kopp (p-kopp@northwestern.edu)

*Thyroid Research 2023*, **16(S1):**S4

Numerous contributions have enriched our knowledge in various fields of thyroidology during the last year. They include, for example:

The full-length structure of the thyroid-stimulating hormone receptor (TSHR) in complex with TSH or antibodies reveals that activation is mediated by an upward (“push and pull”) movement of the extracellular domain.

The structural characterization of the sodium-iodide symporter NIS provides a mechanistic understanding how NIS selects, couples, and translocates iodide and other anions.

Structural information has been generated for human thyroid peroxidase (TPO) in complex with TPO antibodies.

Mutations in human transthyretin (TTR) are associated with familial polyneuropathy (TTR-FAP); certain molecules stabilize TTR as confirmed by structural analysis of VCP-6 bound to TTR.

Thyroid organoids have been generated from human embryonic stem cells. After transplantation, they can normalize thyroid hormone levels in athyreotic mice.

In addition to their classic actions, anterior pituitary hormones also mediate central neural functions. Mapping of the mouse brain has identified Tshr transcripts in the ependymal layer of the third ventricle that is similar to that in thyroid follicular cells.

The solute carrier SLC17A4 has been identified as a transporter of thyroxine (T4) and triiodothyronine (T3) that is widely expressed in the gastrointestinal tract.

Using BeWo cells as model system, the thyroid hormone transporters MCT8 and LATS appear to play a key role in T3 uptake in the placenta; the transporter system mediating T4 uptake remains to be identified.

The X-linked Allan-Herndon-Dudley syndrome (AHDS) results in severe psychomotor disability. It is caused by mutations in the thyroid hormone transporter MCT8. Adeno-associated virus-mediated expression of the Mct8 partially can improve the neurological phenotype in mice.

This presentation will succinctly highlight selected research articles, acknowledging that many studies of interest cannot be covered. The overview will illustrate that there are many novel findings that have fundamental and translational relevance.

## S5 Quality of Life in Hypothyroidism

### Colin Dayan

#### Thyroid Research Group, Cardiff University School of Medicine, Cardiff, UK

##### **Correspondence:** Colin Dayan (dayancm@cardiff.ac.uk)

*Thyroid Research 2023*, **16(S1):**S5

Repeated surveys have confirmed a reduction in quality of life in hypothyroidism. With over 1 million people on thyroid hormone in the UK, this affects a lot of people. The reasons are likely to be complex and may not all be related to thyroid hormone levels. For example, coexistent psychological distress might lead to thyroid function testing which reveals asymptomatic subclinical disease and results in the initiation of ineffective thyroid hormone therapy. On the other hand, biological mechanisms exist to explain why replacement with levothyroxine (T4) alone may exacerbate symptoms in hypothyroidism and recent data on the effect of genetic polymorphisms argue for a biologic contribution to the effect. However, the failure of multiple studies of combined T4+T3 therapy to reach statistical significance has again called into question this contribution. Here we will discuss why this might be and how these issues might be resolved.

## S6 Treatment decisions in Graves’ disease

### Caroline van Kinschot

#### Academic Center for Thyroid Diseases, Department of Internal Medicine, Erasmus Medical Center, Rotterdam, the Netherlands

##### **Correspondence:** Caroline van Kinschot (c.vankinschot@erasmusmc.nl)

*Thyroid Research 2023*, **16(S1):**S6

Treatment options for Graves’ disease (GD) consist of antithyroid drugs (ATD), radioactive iodine (RAI) and total thyroidectomy. Large differences in treatment patterns are observed worldwide. Guidelines recommend discussing the treatment options with patients, taking into account clinical features, benefits, risks, logistic aspects, and a patient’s personal values and preferences. The final treatment decision should be the result of a shared decision process with a patient-centered approach. Recently, we have demonstrated that remission rate is the dominant factor in treatment decision making for GD *(van Kinschot et al., Euro J Endocrinol 2021)*. Among types of treatment, ATD is most preferred by both patients and clinicians. When it comes to definitive treatments, clinicians prefer RAI over surgery, while patients prefer surgery over RAI. A subgroup of patients expressed a strong negative preference for RAI treatment. Anxiety about radioactivity seems to be a widespread concern for patients, probably caused by association of radioactivity with nuclear disasters and warfare and fear for an increased cancer risk. Clinicians’ preference for RAI is probably the result of a better understanding of the more advantageous safety profile of RAI compared to surgery and the absence of fear for radioactivity. Clinicians should be aware that their personal attitude towards RAI differs from their patients, and there is room for improvement in patient counselling regarding this subject. Historically, short-term recurrence after a course of ATD prompted treatment with RAI or surgery. Recent studies, however, have shown that continuous low-dose ATD treatment is a safe option. This will obviously not result in remission in the sense of curation, but this approach does remit hyperthyroidism without the complications related to surgery and RAI and might better connect with patients’ preferences. A better understanding of patients’ preferences and concerns will contribute to the shared decision process and may help in the development of decision aids for GD patients.

**Reference**

van Kinschot CMJ, Soekhai VR, de Bekker-Grob EW, Visser WE, Peeters RP, van Ginhoven TM, van Noord C. Preferences of patients and clinicians for treatment of Graves' disease: a discrete choice experiment. Eur J Endocrinol. 2021; 184:803-812.

## OR1 Comparison of Long-term Mortality and Cardiometabolic Effects of Treatment for Hyperthyroidism: EGRET Study

### Barbara Torlinska^1^, Jonathan M. Hazlehurst^1^, Krishnarajah Nirantharakumar^1^, G. Neil Thomas^1^, Julia Priestley^2^, Keith R Abrams^3^, Kristien Boelaert^1^

#### ^1^Institute of Applied Health Research, University of Birmingham, Birmingham, UK; ^2^British Thyroid Foundation, Harrogate, UK; ^3^Department of Statistics, University of Warwick, Coventry, UK

##### **Correspondence:** Barbara Torlinska (b.torlinska@bham.ac.uk)

*Thyroid Research 2023*, **16(S1):**OR1

**Background and Aims:** Hyperthyroidism has been linked to long-term cardiovascular and metabolic morbidity and mortality. Current evidence indicates differential cardio-metabolic effects from antithyroid drugs (ATD) and definitive treatment (radioiodine or thyroidectomy). We assessed differences in mortality and cardiometabolic outcomes depending on treatment modality to better inform patient-clinician decision-making.

**Methods:** We identified 55,318 patients with newly diagnosed hyperthyroidism, treated with ATD, radioiodine or thyroidectomy from GP data in Clinical Practice Research Datalink (CPRD) (>2,000 contributing GPs, >16M patients) linked with Hospital Episode Statistics (HES). Obesity was compared with data from Health Survey for England. All-cause mortality, major cardiovascular events (MACE: cardiovascular death, heart failure or stroke) and post-treatment obesity were studied. Confounding was controlled using inverse-probability weights. Hazard rates were modelled with Cox PH, probabilities with treatment effects and odds ratios with binary regression (NIHR200772).

**Results:** 77.6% of patients were treated with ATD, 14.6% received radioiodine and 7.8% underwent thyroidectomy. The average follow-up was 12.1 years (SD = 5.2). Compared with ATD treated patients, mortality was significantly decreased in those treated with radioiodine (HR 0.87 [0.83-0.92]) or thyroidectomy (HR 0.80 [0.73-0.90]). The estimated risk of MACE if the population were treated with ATD was 10.2% (9.9-10.5), which increased by an additional 1.3% (0.5-2.1; *P* = 0.001) with radioiodine but not with thyroidectomy (0.1% [-1.1, 1.3], *P* = 0.08). Compared with the background population, thyroidectomy was associated with an increased likelihood of obesity in both men (OR=1.57 [1.29-1.91], P<0.0001), and women (1.27 [1.16-1.39], P<0.0001), while radioiodine induced obesity in women (1.13 [1.06-1.20], *P* = 0.0002) but not in men (1.04 [0.93-1.16], *P* = 0.5).

**Conclusion:** EGRET is the first large study using population-based linked community and hospital data to compare the long-term consequences of treatment modalities for hyperthyroidism. We confirmed decreased mortality in patients treated with radioiodine or thyroidectomy. In these patients, a slightly increased risk of obesity was found. Compared to medical treatment, a small increase in cardiovascular events was noted following radioiodine.

## OR2 The relation between thyroid function and lipid metabolomics and response to combination thyroid hormone replacement

### Peter N Taylor^1^, Stephanie Hanna^1^, Adrian Sayers^2^, Robert French^1^, Bjørn Asvold, LDKE Premwardhana^1^, Michael Stedman^3^, Aled Rees^4^, Onyebuchi Okosieme,^1^ Nicholas Timpson^2^, Adrian Heald^5^ Colin M Dayan^1^

#### ^1^Thyroid Research Group, Systems Immunity Research Institute, Cardiff University School of Medicine, Cardiff, UK; ^2^Department of Social and Community Medicine, University of Bristol, Bristol, UK; ^3^Res Consortium; ^4^Neuroscience and Mental Health Research, Cardiff University School of Medicine, Cardiff, UK; ^5^University of Manchester, Manchester UK

##### **Correspondence:** Peter Taylor (taylorpn@cardiff.ac.uk )

*Thyroid Research 2023*, **16(S1):**OR2

**Introduction:** Thyroid hormones are essential for maintaining metabolic balance and particularly influence lipid synthesis and degradation. Metabolomics and in-depth lipid profiling may enable us to assess for differential effects of thyroid hormone and provide insight into tissue thyroid exposure.

**Methods:** 4,347 children from the Avon Longitudinal Study of Parents and Children (ALSPAC) who had thyroid function/plasma NMR metabolomics measured at age 7 were studied. A subset also had repeat thyroid function/metabolomics performed at age 15 (*N* = 1,811). Linear regression was performed to assess the association between thyrotropin, free tri-iodothyronine (FT3) or free thyroxine (FT4) on metabolite levels. Analyses were adjusted for sex/BMI. We then studied 542 individuals from the WATTS trial where individuals were randomised to receive combination thyroid hormone replacement (liothyronine + levothyroxine) or standard levothyroxine to compare selected metabolites and response to treatment as assessed by quality-of-life measures (GHQ/HADS).

**Results:** Multiple associations after correction for multiple testing were observed for thyrotrophin, FT3 and FT4 on metabolites (P<0.001). Most robust/consistent associations were observed for FT3. The classic inverse association between thyrotrophin and free thyroid hormones was often not observed. The strongest 29 metabolite associations for FT3:FT4 ratio were then studied in the WATTS trial. In individuals on liothyronine and levothyroxine 8 metabolites were associated with higher quality of life (QOL) as measured by GHQ and 9 metabolites were associated with lower HADS depression. No associations were seen with QOL scores in individuals on LT4.

**Conclusion:** Modest changes in thyroid hormone especially FT3 have substantial effect on metabolites, that is not sufficiently captured by thyrotrophin alone. Several markers are also associated with higher QOL in individuals on combination thyroid hormone replacement but not in those on levothyroxine monotherapy. This has key implications for monitoring in hypothyroidism/thyroid hormone replacement, suggesting that some measure of tissue level thyroid hormone action would be helpful in assessing adequacy of replacement.

## OR3 Rational drug design of VCP inhibitors to enhance NIS function in radioiodide therapy

### Ling Zha^1^, Katie Brookes^1^, Jana Kim^2^, Benjamin Small^3,4^, Merve Kocbiyik^1^, Selvambigai Manivannan^1^, Hannah R. Nieto^1^, Giovanni Bottegoni^5,6^, Liam R. Cox^7^, Vinodh Kannappan^3,4^, Weiguang Wang^3,4^, Kavitha Sunassee^2^, Philip J. Blower^2^, Vicki E. Smith^1^, Martin L. Read^1*^, Christopher J. McCabe^1^

#### ^1^Institute of Metabolism and Systems Research, University of Birmingham, Birmingham, UK; ^2^School of Biomedical Engineering & Imaging Sciences, King’s College London, London, UK; ^3^Research Institute in Healthcare Science, Faculty of Science and Engineering, University of Wolverhampton, Wolverhampton, UK; ^4^Disulfican Ltd, University of Wolverhampton Science Park, Wolverhampton, UK; ^5^Institute of Clinical Sciences, University of Birmingham, Birmingham, UK; ^6^Università degli Studi di Urbino Carlo Bo, Urbino, Italy; ^7^School of Chemistry, University of Birmingham, Birmingham, UK

##### **Correspondence:** Martin Read (m.l.read.20@bham.ac.uk)

*Thyroid Research 2023*, **16(S1):**OR3

**Background:** New approaches are required to improve the efficacy of drugs capable of enhancing ablative radioiodide (RAI) uptake, especially in RAI-refractory disease. Our recent experiments revealed that valosin-containing protein inhibitors (VCPi) such as clotrimazole and disulfiram markedly increase sodium iodide symporter (NIS) activity in vitro. However, novel drug design strategies may be needed to overcome the poor solubility and rapid metabolism of VCPi which diminish their thyroidal impact in vivo.

**Aim:** To determine whether rational drug design and reformulation strategies improve the efficacy of VCPi on NIS function in vitro and in vivo.

**Methods:** Computational iterative drug design was accompanied by de novo drug synthesis. RAI (^125^I) uptake assays were used to monitor NIS function in vitro. Technetium-99m pertechnetate (^99m^Tc) uptake after intravenous administration was used to evaluate NIS function in wild-type BALB/c mice.

**Results:** Based on 3D modelling and iterative drug construction, we designed 21 novel analogues of clotrimazole and the allosteric VCPi UPDC30425, all with improved predicted bioavailability (LogP), and synthesised 4 additional compounds based on CryoEM high-resolution features of the VCPi NMS873 docking to VCP. In parallel, we prepared albumin nano-encapsulated copper-diethyldithiocarbamate [Cu(DDC)_2_-alb] - a stabilised reformulation of a disulfiram metabolite. While several clotrimazole analogues specifically increased RAI uptake, the greatest impact was observed with Cu(DDC)_2_-alb treatment in thyroidal TPC-1-NIS (2.8-fold; *P* < 0.01) and 8505C-NIS cells (3.0-fold; *P* < 0.01). Importantly, intraperitoneal administration of Cu(DDC)_2_-alb significantly induced thyroidal ^99m^Tc-uptake in BALB/c mice (~40%; *n *= 11;3mg/kg dose; *P* < 0.001), as well as increasing thyroidal NIS (1.9-fold; *P* < 0.01) and thyroid peroxidase mRNA (1.8-fold;P<0.001) expression. A significant positive correlation was apparent between thyroidal ^99m^Tc-uptake and NIS mRNA (r_s_=0.4477; *P* = 0.0169) in Cu(DDC)_2_-alb treated mice, uniting systemic drug effects on NIS expression and function.

**Conclusions:** Our study demonstrates a promising drug strategy utilising a disulfiram metabolite to enhance NIS function in vivo, with clinical potential to improve treatment in RAI-refractory thyroid cancer.

## OR4 Exploiting endocytic factors as druggable targets to enhance sodium iodide symporter activity with clinical implications for radioiodide therapy

### Katie Brookes^1^, Ling Zha^1^, Jana Kim^2^, Merve Kocbiyik^1^, Selvambigai Manivannan^1^, Kavitha Sunassee^2^, Philip J. Blower^2^, Hannah R. Nieto^1^, Vicki E. Smith^1^, Martin L. Read^1^, Christopher J. McCabe^1^

#### ^1^Institute of Metabolism and Systems Research, University of Birmingham, Birmingham, UK; ^2^School of Biomedical Engineering & Imaging Sciences, King’s College London, London, UK

##### **Correspondence:** Katie Brookes (k.baker.2@bham.ac.uk)

*Thyroid Research 2023*, **16(S1):**OR4

**Background:** Suboptimal radioiodide (RAI) treatment is frequently associated with diminished targeting and retention of the sodium iodide symporter (NIS) at the plasma membrane (PM). The mechanisms which govern the endocytosis of NIS away from the PM are however ill-defined and may have therapeutic potential. We recently demonstrated that NIS internalisation was modulated by the interaction of a C-terminal diacidic motif with the heterotetramer Adaptor Protein 2 (AP2) – a key regulatory factor in endocytosis. Here, we determined whether NIS endocytosis represents a druggable process to enhance RAI uptake.

**Methods:** PM localisation of NIS was quantified via NanoBRET and cell surface biotinylation assays (CSBA). RAI (^125^I) uptake assays were used to monitor NIS function *in vitro*. Intravenous technetium-99m pertechnetate (^99m^Tc) uptake was used to evaluate NIS function in wild-type BALB/c mice.

**Results:** The drug chloroquine (CQ) rapidly increased ^125^I uptake in TPC1-NIS (2.54-fold; P<0.0001) and 8505C-NIS (1.93-fold; P<0.05) cells peaking after 8 hr, which was abrogated by co-treatment with the endocytosis inhibitor Dynasore. Subsequent CSBA confirmed elevated levels of cell-surface NIS in CQ-treated thyroid cancer cells. This finding was supported in live CQ-treated cells via KRAS-NanoBRET assays, where CQ gave a strong BRET signal similar to Dynasore, suggesting that NIS was retained at the PM. To challenge this, we ablated PICALM, an endocytic factor known to recruit AP2/clathrin to the PM which prevented significant induction of RAI uptake by CQ. *In vivo*, CQ treatment of BALB/c mice significantly enhanced thyroidal ^99m^Tc-uptake in combination with the HDACi SAHA (52.7%; *n* = 10; *P* = 0.0003), as well as increasing thyroidal expression of NIS (2.2-fold; *P* < 0.0001), TSHR (1.9-fold; *P* = 0.001) and PAX8 mRNA (1.6-fold; *P* = 0.003).

**Conclusions:** Our findings suggest that CQ interferes with the PICALM/AP2/clathrin machinery which controls NIS endocytosis, identifying it as an FDA-approved pharmaceutical agent which alters NIS endocytosis, with translatable potential to improve radioiodide therapy in thyroid cancer.

## OR5 NOX3 Variant Effect on Anti-Thyroid Drug-Induced Agranulocytosis (ATD-Ag)

### Rachel E Holliday,^1^ Kath Allinson,^1^ Will Drake,^2^ Krishna Chatterjee,^3^ Amit Allahabadia,^4^ Kristien Boelaert,^5^ Salman Razvi,^6^ Colin Dayan,^7^ Florian Wernig,^8^ Onyebuchi Okosieme,^9^ Jackie Gilbert,^10^ Ahmed Al-Qaissi,^11^ Tristan Richardson,^12^ Anastasios Gazis,^13^ Zin Zin Htike,^13^ Moulinath Banerjee,^14^ Vladmir Vaks,^15^ Christine May,^16^ Rupa Ahulwalia,^17^ James McClaren,^18^ Prakash Abraham,^19^ Graham Leese,^20^ Nidhi Choudhary,^21^ Simon H Pearce,^1^ Bijay Vaidya^22^

#### ^1^Newcastle University, Newcastle upon Tyne; ^2^Barts Health NHS Trust, London; ^3^Cambridge University Hospitals NHS Foundation Trust, Cambridge; ^4^Sheffield Teaching Hospital NHS Foundation Trust, Sheffield; ^5^Birmingham University, Birmingham; ^6^Gateshead Health NHS Foundation Trust, Gateshead; ^7^Cardiff University, Cardiff; ^8^Hammersmith Hospital, London; ^9^Cwm Taf Morgannwg University Health Board, Pontypridd; ^10^King's College Hospital, London; ^11^St James’ University Hospital, Leeds; ^12^University Hospitals Dorset NHS Foundation Trust, Bournemouth; ^13^Nottingham University Hospital, Nottingham; ^14^Bolton Hospital, Bolton; ^15^Great Western Hospital, Swindon; ^16^Oxford University Hospitals NHS Foundation Trust, Oxford; ^17^Norfolk & Norwich University Hospitals NHS Foundation Trust, Norwich; ^18^University Hospital Wishaw, Wishaw; ^19^Aberdeen Royal Infirmary, Aberdeen; ^20^Ninewells Hospital, Dundee; ^21^Derriford Hospital, Plymouth; ^22^University of Exeter, Exeter

##### **Correspondence:** Rachel Holliday (r.e.holliday@newcastle.ac.uk)

*Thyroid Research 2023*, **16(S1):**OR5

**Background**: Agranulocytosis is a rare, life-threatening side effect of anti-thyroid drugs (ATD-Ag). Although a pharmacogenomic basis is suspected, it has not currently been defined in detail. A whole exome sequencing study of 11 Dutch ATD-Ag cases (including 5 from 2 families) identified a missense variant in the NADPH oxidase-3 (NOX3) gene (Ala198Thr; rs117412760), that was present in 3 of 8 (37%) unrelated ATD-Ag patients and which co-segregated with ATD-Ag in one family *(Plantinga et al., 2017)*. This NOX3 variant is present in only 2.1% of European individuals, suggesting a significant enrichment in ATD-Ag cases.

**Aim**: Our study sought to determine whether the association of this NOX3 gene variant, rs117412760, with ATD-Ag would be reproducible in a larger patient cohort.

**Method:** 51 ATD-Ag cases were identified by the Society for Endocrinology UK ATD study and genomic DNA was extracted using sodium perchlorate/chloroform, along with control DNA for 151 UK Graves’ disease patients who had never had ATD-Ag. An allele discrimination TaqMan assay for rs117412760 was used to test the full cohort of 51 UK ATD-Ag patients and 151 controls. The Taqman QS7 PCR machine was used to genotype and plot the genotypes. 18/151 (12%) sample controls were repeated in duplicate, and assay reproducibility was 100%.

**Results:** The genotyping results showed that the NOX3 Ala198Thr variant was found in none of the 51 (0%) ATD-Ag patients but in 5 of the 151 (3.3%) Graves’ controls. Analysis according to allele frequencies showed no significant difference (*p* = 0.34; Fisher exact test).

**Conclusion:** Contrary to the Dutch study, these results strongly suggest that this NOX3 variant does not contribute to the susceptibility to antithyroid drug induced agranulocytosis. Further genetic analysis will now be undertaken.

## L2 BTF Research Award Update: Management of hyperthyroidism during pregnancy: data from a large primary care cohort

### Peter N Taylor^1^, Bijay Vaidya^2^

#### ^1^Thyroid Research Group, Systems Immunity Research Institute, Cardiff University School of Medicine, Cardiff, UK; ^2^Royal Devon University Hospital & University of Exeter, Exeter, UK

##### **Correspondence:** Peter Taylor (taylorpn@cardiff.ac.uk )

*Thyroid Research 2023*, **16(S1):**L2

Hyperthyroidism is common and occurs frequently in women of child-bearing age. Hyperthyroidism during pregnancy is associated with adverse outcomes, anti-thyroid drugs have teratogenic potential and women who have had definitive treatment for hyperthyroidism usually need levothyroxine which requires additional monitoring. At present it is unclear how preconception treatment strategies in women with established hyperthyroidism impact on thyroid status in subsequent pregnancy.

Broadly there are 3 treatment scenarios, (1) antithyroid drugs up to or beyond onset of pregnancy, (2) definitive treatment with thyroidectomy or radioiodine before pregnancy, and (3) no treatment at pregnancy onset. We sought to investigate trends in hyperthyroidism management and which of these treatment strategies resulted in increased odds of having sub-optimal thyroid status during pregnancy (TSH >4.0 mU/L or TSH <0.1 mU/L plus FT4 >reference range). We therefore utilised the Clinical Practice Research Datalink (CPRD) database to evaluate all females aged 15-45 years, with a clinical diagnosis of hyperthyroidism and a subsequent pregnancy record between January 2000 to December 2017 *(Minassian et al., J Clin Endocrinol Metab 2023)*.

We identified 4,712 pregnancies with a prior diagnosis of hyperthyroidism. Between 2000-2008, the prevalence of radioiodine before pregnancy fell from 4.7% to 2.9%, returning to 4.7% by 2011, then dropping further to 1.8% by 2017. The prevalence of thyroid surgery before pregnancy decreased more steadily over the study period, from 19.0% to 11.5% (2000-2017). Women treated with radioiodine or thyroid surgery before pregnancy were less likely to have TSH or FT4 recorded during pregnancy than women who became pregnant on antithyroid drugs (65.3% versus 73.8%).

Overall, 837 pregnancies (17.8%) were exposed to antithyroid drug treatment. Between 2000-2011, the proportion of pregnancies prescribed Carbimazole fell from 10.9% to 8.6%, then gradually increased to 11.5% by 2017. A reverse pattern was seen for PTU. Pregnancies with prior radioiodine or thyroid surgery had markedly increased odds of having suboptimal thyroid status compared to pregnancies starting during antithyroid drug treatment (OR 4.48, 95% CI 3.31-6.06).

Overall, our data shows that the management of women with hyperthyroidism who become pregnant is suboptimal and needs urgent improvement.

**Acknowledgements**

We are grateful to receive the award from the British Thyroid Foundation and assistance from Caroline Minassian, LSHTM for data analysis.

**Reference**

Minassian C, Allen LA, Okosieme O, Vaidya B, Taylor P. Preconception management of hyperthyroidism and thyroid status in subsequent pregnancy: a population-based cohort study. J Clin Endocrinol Metab. 2023 May 18:dgad276.

## PO1 Diverse Clinical and Laboratory Phenotypes associated with Heterozygous *PAX8* Mutations

### V Ravikumar^1^, C Peters^2^, M Cerbone^2^, VB Arya^3^, P Agrawal^3^, H Katugampola^2^, S Langham^2^, E Schoenmakers^1^, N Schoenmakers^1^

#### ^1^Wellcome Trust-MRC Institute of Metabolic Science, University of Cambridge, Cambridge, UK; ^2^Great Ormond Street Hospital for Children, London, UK; ^3^King’s College Hospital NHS Foundation Trust, London, UK

##### **Correspondence:** Nadia Schoenmakers (naaa2@cam.ac.uk)

*Thyroid Research 2023*, **16(S1):**PO1

**Background and Aims:** The transcription factor Paired box gene 8 (PAX8) is required for thyroid development and maintenance of the differentiated thyroid phenotype, mediating transcriptional activation of key thyroidal genes, and synergizing with NKX2-1 at the *TG* promoter. Heterozygous *PAX8* mutations cause congenital hypothyroidism (CH) with variable thyroid morphology and biochemical severity (classically thyroid hypoplasia), and occasional urogenital tract malformations. We delineate clinical phenotypes in 4 kindreds harbouring heterozygous PAX8 mutations, and characterize the mutant PAX8 proteins in vitro.

**Method:** Clinical data was acquired as part of routine care; *PAX8* was screened as a candidate gene. Laboratory studies were performed in transfected heterologous cells.

**Results:**

p.S59R, p.R207* and p.I34F demonstrated impaired transactivation of *TPO* and *TG* promoters in luciferase reporter assays, compared to wild-type PAX8. p.S54R has previously been well-characterized with similar results (1). Co-transfection with NKX2-1 at least partially rescued *TG* promoter transactivation with p.I34F and p.S59R but not p.R207*, supporting a role for the PAX8 carboxyterminus in this interaction. Homology modelling suggests steric hindrance due to p.S59R and p.I34F mutations may affect PAX8-DNA interactions.

**Conclusions:** Our studies characterize a novel PAX8 mutation (p.I34F) associated with thyroid and urogenital tract pathology, and yield further insights into PAX8-NKX2-1 synergism. Additionally, we expand the clinical phenotypes associated with reported PAX8 mutations, demonstrating isolated CH and possible impaired SLC5A5 function with p.R207*, and thyroid hemiagenesis with p.S59R.

**Reference**

1. Hermanns (2013) Thyroid 23(7):791–6.

**Table 1:** See text for description

Case	Phenotype	PAX8 Mutation	Previous associations
P1	Severe CH, thyroid hypoplasia, hypospadias	p.I34F	Novel
P2	Mild CH, absent Tc-99m uptake in a normal sized thyroid, normal urogenital tract	p.R207*	Previously reported in Mayer-Rokitansky-Kuster-Hauser (MRKH) syndrome
P3	Severe dysgenic CH	p.S54R	Previously reported in thyroid dysgenesis
P4a, P4b (sisters)	Permanent gland-in-situ CH Mild CH with hemiagenesis	p.S59R	Previously associated with severe gland-in-situ CH, or goitrous CH with cryptorchidism and hydrocele

## PO2 Elucidating the mechanisms via which the proto-oncogene PBF stimulates cell motility in thyroid tumour progression

### Selvambigai Manivannan^1^, Merve Kocbiyik^1^, Mohammed Alshahrani^1^, Mohammed E El-Asrag^2^, Ling Zha^1^, Katie Brookes^1^, Hannah R Nieto^1^, Martin L Read^1^, Andrew Beggs^2^, Chris J McCabe^1^, Vicki E Smith^1^

#### ^1^Institute of Metabolism and Systems Research (IMSR), University of Birmingham, UK; ^2^Institute of Cancer and Genomic Sciences (ICGS), University of Birmingham, UK

##### **Correspondence:** Selvambigai Manivannan (s.manivannan@bham.ac.uk)

*Thyroid Research 2023*, **16(S1):**PO2

**Background and Aims:** The proto-oncogene pituitary tumor-transforming gene binding factor (PBF/PTTG1IP) is overexpressed in multiple tumours, including thyroid cancer, and is associated with tumour progression. In vitro, PBF potently induces cancer cell motility, and both Src-mediated phosphorylation at tyrosine 174 (Y174) and endocytosis of PBF are required for induction of thyroid and breast cancer cell migration and invasion. This study aimed to further elucidate the mechanisms by which PBF induces cancer cell motility.

**Methods:** To elucidate molecular events downstream of PBF overexpression, phosphoproteomic and RNA-Seq analyses of thyroid cells stably overexpressing PBF were performed. We then utilised a novel Pbf knockout (Pbf^-/-^) mouse model with CRISPR/Cas9-mediated deletion of Pbf exon 4 in C57BL/6N mice. Mouse embryonic fibroblasts (MEFs) were isolated at embryonic day 13.5 and used as primary cultures.

**Results:** Phosphoproteomic and RNA-Seq analyses revealed enrichment for molecules involved in cell adhesion and cytoskeleton organisation in response to PBF overexpression, prompting further investigation into a physiological role for PBF in cell motility. Pbf^-/-^ MEFs showed a significant reduction in migration and invasion compared with wild-type (Pbf^+/+^) MEFs. Interestingly, heterozygote MEFs (Pbf^+/-^) showed an intermediate decrease in motility suggesting a gene-dosage effect. Additionally, the migration of Pbf^-/-^ MEFs transfected with PBF was comparable with Pbf^+/+^ MEFs in rescue experiments. Initial immunofluorescent studies of Pbf^-/-^ MEFs suggest alterations in focal adhesion (FA) structure and distribution. Importantly, Pbf^-/-^ MEFs demonstrated a marked reduction in focal adhesion kinase (FAK) and paxillin staining with smaller, punctate and more radially distributed FAs compared with Pbf^+/+^ MEFs.

**Conclusions:** These findings further confirm a role for PBF in cell motility, through regulating cell adhesion and cytoskeleton organisation. Overall, these studies demonstrate a physiological role for PBF in cell motility and further elucidate the mechanisms by which PBF induces cell motility in thyroid tumour progression.

## PO3 Iopanoic acid for pre-operative preparation for total thyroidectomy in patients with thyrotoxicosis: a case series

### Christine Newman, Muhammed Saqlain, Isra Ahmed Mohammed, Daniel Bell, Nadia Schoenmakers, Carla Moran, Diana Woods, Krishna Chatterjee

#### Wolfson Diabetes and Endocrinology, Addenbrooke’s Hospital, Cambridge University Hospitals NHS Foundation Trust, Cambridge, UK

##### **Correspondence:** Christine Newman (newman.christine17@gmail.com)

*Thyroid Research 2023*, **16(S1):**PO3

**Background and Aims:** Iopanoic acid (IOPA) is a safe, previously widely-available, oral cholecystographic contrast agent used to control hyperthyroidism. Uniquely, this iodine-containing contrast agent is metabolised via the hepatobiliary system, enabling blockade of hepatic type I deiodinase thereby inhibiting T4 to T3 conversion. Newer gallbladder imaging modalities have led to cessation of its manufacture. We reviewed clinical and biochemical data from a series of patients treated with pre-operative IOPA due to intolerance to or failure to respond to other therapies.

**Methods:** With prior permission of our Drug & Therapeutics Committee, we sourced laboratory-grade (99% pure) IOPA and administered this preoperatively to control life-threatening hyperthyroidism, not responding to other therapies (Lugol’s iodine, steroids, high-dose thionamides) in patients with amiodarone-induced thyrotoxicosis (AIT) (*n* = 5), Graves’ disease (*n* = 4), toxic multinodular goitre (*n* = 1) and resistance to thyroid hormone beta (*n* = 1). Patients were treated between 2014-2023.

**Results:** All patients had significant thyrotoxicosis at baseline (mean fT4 59.4 mU/L (range 10.5-21), mean fT3 12.1 mU/L (3.5-6.5)) and were treated with IOPA for an average of 6 days before urgent/emergency thyroidectomy. FT3 declined in all and normalised within 42hrs in 8/11. Reduced platelet counts in 3 cases (103, 105 and 92 x10^9^/L) predated IOPA therapy and were likely related to coexistent pulmonary hypertension and thyrotoxicosis. IOPA was well tolerated with no gastrointestinal side effects or deterioration in liver function; renal function remained normal except in one patient who had high-dose frusemide for cardiac failure. A single postoperative death was due to hospital-acquired pneumonia.

**Conclusions:** IOPA is a highly effective and safe agent for controlling biochemical thyrotoxicosis refractory to other agents, as a prelude to thyroid surgery. Highly pure, laboratory-grade iopanoic acid that is approved for human use, is now available in the UK and could be encapsulated for hospital pharmacies to stock this agent for emergency use in uncontrolled, life-threatening hyperthyroidism.

## PO4 British Thyroid Association survey of Graves’ disease management in the UK

### Koteshwara Muralidhara^1^, Prakash Abraham^2^, Bijay Vaidya^3^, Onyebuchi Okosieme^4 5^

#### ^1^Kingston Hospital NHS Foundation Trust; ^2^Endocrine Unit, David Anderson Building, Aberdeen Royal Infirmary, Forester hill Road; ^3^Royal Devon & Exeter Hospital, University of Exeter Medical School, Exeter EX2 5DW; ^4^Cwm Taf Morgannwg Health Board, Merthyr Tydfil, UK; ^5^Thyroid Research Group, Systems Immunity Research Institute, Cardiff University School of Medicine, UK

##### **Correspondence:** Onyebuchi Okosieme (okosiemeoe@cardiff)

*Thyroid Research 2023*, **16(S1):**PO4

**Background:** The current state of practice in the management of Graves’ hyperthyroidism in the UK is unclear. The impact of recent NICE thyroid disease guidelines together with post-pandemic resource challenges are still unravelling. The survey aimed to understand current approaches in Graves’ disease management amongst UK endocrinologists.

**Method:** Between October 2022 and March 2023, endocrinologists were invited to complete a 10-minute online survey, circulated by e-mail link to members of the British Thyroid Association (BTA), the Society for Endocrinology (SfE), and regional endocrinology networks.

**Results:** Out of 160 eligible respondents, majority were endocrinologists (99%) and consultants (91%). For a 40-yr-old female with a first presentation of Graves’ hyperthyroidism, majority (93%) would treat with antithyroid drugs (ATD) and request TSH receptor Antibodies (TRAb) at diagnosis (95%) and at follow-up (76%). RAI was preferred for primary therapy by 3%, and for recurrent Graves’ disease by 81% of respondents. Almost all (97%) respondents preferred Carbimazole (CMZ) which was used for 12-18 months (70%). Propylthiouracil (PTU) was the drug of choice in early pregnancy (84%), and after the first trimester, 50% of respondents continued PTU in women on PTU while 50% switched back to CMZ.

**Conclusions:** ATDs remain the preferred first-line therapy for Graves’ disease with an increasing number of endocrinologists using TRAbs for diagnosis as well as prognosis. PTU is the preferred ATD in early pregnancy but treatment choice after the first trimester remains divided. Aspects of current practice deviate from guidelines probably driven by resource availability and physician preference.

## PO5 Exploring the association between thyroid nodules location and the risk of malignancy: findings from a pilot retrospective study

### Bilal Jajah, Thazin Wynn, Jayamalee Jayaweera, James Maye, Rogers Rebecca, Khin Pyae Soe, Finch Laurie

#### West Suffolk Hospital, Bury St Edmunds, UK

##### **Correspondence:** Bilal Jajah (drmbilal77@gmail.com)

*Thyroid Research 2023*, **16(S1):**PO5

**Background:** Some studies have suggested that thyroid nodules located in the isthmus or upper pole of the thyroid lobe may have a higher risk of malignancy, but this relationship is not well understood. This pilot retrospective study aimed to investigate the association between thyroid nodule location and malignancy risk.

**Methods:** We conducted a retrospective review of medical records for all patients who underwent fine needle aspiration biopsy at West Suffolk Hospital between 2016-2022. Nodules were categorized as benign or malignant based on confirmed histology or cytology results. Thyroid nodule location was determined by reviewing the histopathology report, when available, and by analyzing the longitudinal ultrasound view when histopathology information was not provided.

**Results:** The study included 317 thyroid nodules, among which those occupying the entire thyroid lobe had a higher frequency of malignancy (44%, odds ratio [OR] 2.67, 95% confidence interval [CI] 1.43 to 5.10) compared to nodules in other regions. While nodules in the upper (36%, OR 1.84, 95% CI 0.76-4.34) and middle (33%, OR 1.44, 95% CI 0.71 to 2.95) lobes also had a higher frequency of malignancy compared to the lower lobe, this result was not statistically significant. No significant difference in malignancy frequency was observed between the right and left lobes.

**Conclusion:** Our Study showed that the nodules occupying the entire thyroid lobe had a significantly higher frequency of malignancy, which could potentially be used to improve the accuracy of existing US risk stratification systems. However, we did not observe a significant association between malignancy risk and nodules located in the upper pole or isthmus of the thyroid gland, in contrast to some previous reports. This discrepancy may be due to the small sample size in our study. Further studies with larger sample sizes are needed to confirm these findings and explore the potential clinical implications.

## PO6 Treatment resistant Amiodarone-induced Thyrotoxicosis in a patient with laminopathy requiring salvage thyroidectomy

### DJ Tansey^1 2^, J O’Connell^3^, R McQuillan^3,4^, S Frohlich^5^, M Griffin^2,6^, J Lyne^4^, C Moran^1 2 6^

#### ^1^St. Vincent’s University Hospital, Elm Park, Dublin 4, Ireland; ^2^Department of Endocrinology and Diabetes Mellitus, Beacon Hospital, Sandyford, Dublin 18, Ireland; ^3^Blackrock Clinic, Dublin; ^4^Beacon Hospital, Dublin; ^5^ Intensive care Unit, Beacon Hospital, Dublin; ^6^UCD School of Medicine, University College Dublin, Belfield, Dublin 4, Ireland

##### **Correspondence:** David Tansey (drdavidjtansey@gmail.com)

*Thyroid Research 2023*, **16(S1):**PO6

**Background:** Amiodarone induced thyrotoxicosis (AIT) is a potentially catastrophic situation for patients with cardiac disease who are at risk of life-threatening complications. We describe the protracted and challenging journey of a patient with Laminopathy and a significant cardiac history who developed AIT.

**Clinical Case:** A 53-year-old man was referred with thyrotoxicosis (TSH < 0.01mU/L, free T4 61 pmol/L, free T3 8.0 pmol/L) detected following commencement of amiodarone for atrial fibrillation. He had a history of laminopathy (due to *LMNA* mutation), associated cardiomyopathy and intractable arrhythmia, requiring ICD insertion, atrial and ventricular ablations. Medications included Amiodarone, Mexiletine, Bumetanide, Eplerenone, Bisoprolol, Entresto, Rivaroxaban, and Empagliflozin. Laminopathy was present in numerous relatives, including his mother, who had a history of AIT also. TRAb antibody was < 0.8 IU/L. An ultrasound thyroid showed normal sized gland, mildly heterogenous echogenicity and reduced vascularity.

He was treated with carbimazole and dexamethasone for AIT but remained thyrotoxic, and developed cardiac decompensation (oedema, SOB) with early myopathy. Second line agents cholestyramine and iodine solution were added, without effect. It was decided that he required salvage thyroidectomy, and he underwent numerous plasma exchanges in preparation for surgery. Following thyroidectomy, the patient recovered well. His thyroid and cardiac symptoms improved, and he was discharged on Eltroxin with follow up.

**Conclusion:** We describe a rare case of treatment resistant AIT. The case was complicated by the underlying laminopathy, which further increases the risk of myopathy, cardiac arrhythmia and cardiac decompensation in a thyrotoxic patient. Prompt recognition of treatment resistance is required, because the window of opportunity for safe thyroidectomy in such patients is narrow. Options for rendering patients euthyroid in resistant AIT include perchlorate, iopanoic acid, and therapeutic plasma exchange.

The patient has given consent for publication of this abstract.

## PO7 “It’s a Grave problem”- A case series of hypercalcaemia due to thyrotoxicosis

### Nadia Osman, Henna Patel, Gadintshware Gaoatswe

#### Department of endocrinology and diabetes, Newham University Hospital, Barts Health NHS Trust

##### **Correspondence:** Nadia Osman (n.osman3@nhs.net)

*Thyroid Research 2023*, **16(S1):**PO7

**Background and aims:** Hypercalcaemia is a common clinical problem. It can occur due to the imbalance between calcium entry into the circulation, deposition in bone and urinary excretion. Primary hyperparathyroidism and malignancy account for more than 90% of these cases^1^. Excess thyroid hormones, as occurs in hyperthyroidism can impact calcium metabolism and lead to hypercalcaemia in up to 20% of cases^1^. We describe a case series in which hypercalcaemia occurs alongside concomitant hyperthyroidism. We investigated the effects of antithyroid treatment on serum calcium levels in these patients and review the literature.

**Methods:** We prospectively followed calcium and thyroid hormone levels following commencement of antithyroid medications in three patients presenting with hyperthyroidism and PTH-independent hypercalcaemia. All 3 patients presented with classical symptoms of hyperthyroidism with a constellation of symptoms including weight loss, palpitations, and heat intolerance.

**Results:** All were found to be thyrotoxic with positive TSH receptor antibodies confirming Grave’s disease and thyroxine level was above the assay detection level (100pmol/L). All patients also demonstrated PTH-independent hypercalcaemia, with corrected calcium levels ranging between 2.69 to 3.30mmol/L and required admission for intravenous fluid rehydration. Commencement on a combination of Propranolol and Carbimazole resulted in normalisation of calcium and PTH as thyroxine levels improved.

**Conclusion:** Hypercalcaemia is not uncommon in hyperthyroidism. Thyroid hormones can affect bone metabolism by causing increased bone turnover and accelerated bone remodelling via direct or indirect effects on osteoclast activity^2,3^. As a result, hypercalcaemia occurs independently of PTH. Multiple cases of hyperthyroidism with hypercalcaemia have been reported. In many of these cases, treatment of hypercalcaemia required calcium lowering therapy including calcitonin and bisphosphonates^4^. However, our cases demonstrate the normalisation of serum calcium with no adverse outcomes using antithyroid medications alone. This suggests that early identification and treatment of hyperthyroidism with antithyroid medications may be sufficient in uncomplicated hypercalcaemia due to hyperthyroidism.

All patients have been consented for publication of this abstract.

**References**

1. Shane E. UpToDate. Etiology of hypercalcaemia. Available at: https://www.uptodate.com/contents/etiology-of-hypercalcemia [Accessed 20/3/23].

2. Britto, J. M., Fenton, A. J., Holloway, W. R., & Nicholson, G. C. (1994). Osteoblasts mediate thyroid hormone stimulation of osteoclastic bone resorption. Endocrinology, 134(1),169–76.

3. Pantazi, H., & Papapetrou, P. D. (2000). Changes in parameters of bone and mineral metabolism during therapy for hyperthyroidism. The Journal of clinical endocrinology and metabolism, 85(3), 1099–1106.

4. Chen, K., Xie, Y., Zhao, L., & Mo, Z. (2017). Hyperthyroidism-associated hypercalcemic crisis: A case report and review of the literature. Medicine. 96(4), e6017.

## PO8 A Unique Case of Concomitant Thyroid Paraganglioma and Parathyroid Carcinoma

### Thazin Wynn, Bilal Jajah, Joohi Majeed

#### Diabetes and Endocrinology Department, West Suffolk Hospital NHS Foundation Trust, Bury St Edmunds IP33 2QZ, UK

##### **Correspondence:** Thazin Wynn (t.wynn@doctors.org.uk)

*Thyroid Research 2023*, **16(S1):**PO8

**Background/Aims:** Thyroid paragangliomas are neuroendocrine tumours that arise from the inferior laryngeal paraganglia and account for <0.01% of all thyroid neoplasms. In this report, we present a highly unusual case of a patient with both thyroid paraganglioma and secretory parathyroid carcinoma.

**Case Presentation:** A 49-year-old lady presenting with generalised myalgia was found to have a parathyroid-dependent severe hypercalcaemia, (adjusted calcium 3.93mmol/L, PTH of 134pmol/L). As well as confirming a right parathyroid lesion, neck ultrasound identified an incidental hypoechoic, hypervascular lesion in the left thyroid lobe measuring 2.3x1.6x2.4cm. A core biopsy of the left thyroid lesion was suspicious for paraganglioma. Following a short admission for emergency management of hypercalcaemia, she underwent total thyroidectomy and right parathyroidectomy. Histology confirmed concomitant thyroid paraganglioma and parathyroid carcinoma. MRI whole body did not show further lesions and genetic screening was negative.

**Discussion:** Since 1964, only around 81 cases of thyroid paraganglioma have been reported, and none of these have been in conjunction with parathyroid carcinoma. Diagnosis of thyroid paraganglioma can be easily missed due to their rarity and immunohistological overlap with other thyroid neoplasms. They should be tested for functional status, including serum catecholamines and fasting gut hormones, as well as genetic testing including multiple endocrine neoplasia-1 (MEN-1). Whole-body imaging, such as MRI, should be performed to rule out synchronous paragangliomas.

**Conclusion:** This case highlights the rarity of thyroid paraganglioma and the need for thorough evaluation when encountering such tumours. The concomitant diagnosis of parathyroid carcinoma further adds to the uniqueness of this case. Clinicians should maintain a high level of suspicion for rare tumours such as thyroid paraganglioma, and imaging and histological techniques should be employed as necessary to arrive at a correct diagnosis. Due to the unpredictable nature of these tumours, long-term monitoring for recurrence is necessary.

Written consent to publish had been obtained from the patient.

